# Optimal Voltage for Cranial Electromagnetic Field Stimulation to Modulate Brain Activity

**DOI:** 10.7759/cureus.82011

**Published:** 2025-04-10

**Authors:** Alice S Wang, Paras Savla, James Brazdzionis, Katherine Ko, Dan E Miulli

**Affiliations:** 1 Neurosurgery, Riverside University Health System Medical Center, Moreno Valley, USA; 2 Neurosurgery, Arrowhead Regional Medical Center, Colton, USA

**Keywords:** atraumatic brain injury, constant-voltage stimulation, electromagnetic field stimulation, pulsed electromagnetic field therapy, traumatic brain injury

## Abstract

Background

The electromagnetic field (EMF) patterns of the animal brain with traumatic brain injury (TBI) can be modulated through EMF stimulation. The implication of EMF stimulation is that it can reverse abnormal EMF brain patterns, and this reversal can translate into facilitating a faster clinical recovery. In this paper, the authors applied escalating dosages of voltage stimulation to find the optimal voltage, one that transforms abnormal EMF patterns to normal EMF patterns in patients with atraumatic and traumatic brain injuries.

Methods

EMF data of patients greater than 18 years old diagnosed with brain injury from January 2025 to February 2025 were collected using the DAQami software (DATAQ Instruments, Akron, OH) and analyzed in real time using fast Fourier transform (FFT) with the Igor Pro 8 software (Wavemetrics, Inc., Lake Oswego, OR). EMF stimulation via escalating the dosage of voltage at the targeted frequency of interest (FOI), which was selected based on each patient’s EMF signal related to the clinical presentations and/or radiographic findings, was applied, and clinical and EMF changes were measured in real time.

Results

The mean age was 47.1 years in all 10 patients. Mechanisms of injury included spontaneous hypertensive intracranial hemorrhage (one patient) and head trauma after a motor vehicle collision, dirt bike accident, and ground-level fall (nine patients). Radiographic findings included spontaneous basal ganglia hemorrhage (one patient), isolated traumatic subdural hematoma (one patient), traumatic subarachnoid hemorrhage (one patient), and no intracranial abnormalities (seven patients). Improvement in EMF data was found in two patients after stimulation at 1.0 voltage (V), three patients after stimulation at 3.0 V, one patient after stimulation at 8.0 V, and two patients after stimulation at 10.0 V, and a higher voltage may be needed for two patients with chief complaint of headache and whose EMF data only demonstrated some improvement after stimulation at 10.0 V.

Conclusions

The modulation of the brain at the optimal frequency and voltage stimulation was correlated with improvements in clinical symptoms and signs. The applied EMF frequency was precisely tailored to the identified EMF brain abnormalities of each patient. The escalating dosage of voltage stimulation was followed in real time until the optimal voltage stimulation, one that transformed abnormal EMF patterns into normal EMF patterns, was found. Modulating the brain this way likely reversed the malfunction of molecular and cellular changes and facilitated the recovery of brain functions.

## Introduction

The therapeutic effects of electromagnetic field (EMF) modulation via EMF stimulation have previously been described in animal models with traumatic brain injury (TBI), spinal cord injury, or global transient stroke [1-6]. For instance, EMF stimulation at 2.5 Hertz (Hz) and 5.5 Hz resulted in the improvement of EMF patterns on treatment day 7 in a swine model with induced brain injury [6]. At the cellular level, the EMF stimulation likely induced certain neuronal changes such as gene and protein expression or myelination [1]. In human studies, the EMF patterns in patients with TBI differ from those without TBI such as there are fewer variations in waveforms and fewer peaks and valleys in the former [7]. The differences may be due to a combination of primary and secondary brain injuries associated with TBI. The modulation of the EMF may help counteract the harmful effects of these secondary brain injuries, which are associated with inflammatory cytokines, aberrant cell signaling, and cortical spreading depressions, and thereby facilitate neural recovery [8,9]. The implication of EMF stimulation is that it can reverse abnormal EMF patterns, and this reversal may translate into facilitating a faster clinical recovery.

The stimulation of the brain using pulsed electromagnetic fields (PEMFs) or extremely low-frequency magnetic fields (ELF-MF) is found to induce molecular, cellular, and neuronal changes and provide neuroprotection and reduction in functional deficits in acute ischemic stroke [10-17]. However, current technology, such as PEMF, does not provide continuous, real-time identification of abnormal brain EMF activity from specific areas of the brain, affecting brain function, for which treatment can be tailored to modulate those abnormalities and see the effects of such modulation on EMF recording and clinical assessment in real time. A non-invasive, portable helmet equipped with 20 sensors has been shown to detect, in real time, the electrical and chemical signals that generate EMF [7,18-20]. Previously, the authors investigated the EMF patterns in patients with atraumatic and traumatic brain injuries and were able to localize the site of brain injury and select a specific frequency for stimulation based on certain EMF changes unique to each patient’s clinical symptoms, neurological deficits, and radiographic findings [19,20]. In this paper, the authors investigated how the amplitude or voltage of EMF stimulation changes EMF patterns in real time and can be prescribed in patients with atraumatic and traumatic brain injuries.

## Materials and methods

Study design

Our institution’s (Arrowhead Regional Medical Center) Institutional Review Board approved this prospective study (protocol: #23-58). The inclusion criteria included patients greater than 18 years old with head trauma or intracranial hematomas. The exclusion criteria were as follows: (1) a Glasgow Coma Scale (GCS) of 3, (2) contraindications for donning a helmet such as active hemodynamic or respiratory instability, or (3) the refusal of study enrollment. Two portable racks equipped with horizontal rods and four cords were used to suspend the helmet in the air, and the cords provided adjustable tension to securely hang the helmet in place just above the patient’s head. A portable lightweight helmet with shielding constrained to a dual-layered Mu-metal (MuMETAL, Magnetic Shield Corporation, Bensenville, IL) and copper layering and engineered with Mu-metal 18-inch channels to place sensors and EMF signal generators (BS-1000, Quasar Federal Systems, San Diego, CA) was built to allow for EMF recording at bedside instead of bringing the patient to a room designed for EMF recordings. The sensors and EMF signal generators were placed in a specific configuration. In this paper, each sensor’s spanning region is clarified in parenthesis, for example, sensor 7 (left frontal lobe). The sensors were placed in the channels 9 inches away from the scalp, providing a 6.37-degree field of view. The known spatial relationship between the sensors allowed for identifying regions of overlap or opposite configurations, where sensors in opposing positions (180 degrees from each other) were expected to demonstrate opposite polarities for a specific EMF. Each sensor was also positioned with the positive end oriented toward the scalp.

EMF data collection and analysis

In addition to the detailed methodology described by Wang et al. [19] and Wang et al. [20], the first and second derivatives of pre-stimulation EMF were also analyzed in this study, and the methodology for the derivatives is described by Brazdzionis et al. [7]. Once the localization of brain injuries and frequency selection were achieved, the next step was to apply specific EMF stimulation for the modulation of the neuronal activities and the associated frequencies. EMF signal generators (BS-1000, Quasar Federal Systems, San Diego, CA) were used to deliver various voltage stimulations at the targeted frequency of interest (FOI). A sine wave was used in this study. The stimulation was identified and monitored in real time. The regime for escalating the dosage of voltage stimulation was 1.0 voltage (V), 3.0 V, 5.0 V, 8.0 V, and 10.0 V, each voltage applied over three minutes. Each voltage value had a 50% offset value, either positive or negative, to ensure that the full dynamics of the EMF were captured without the distortion of data. When stimulating at 1.0 V, 3.0 V, 5.0 V, 8.0 V, and 10.0 V, the offset values were half of each amplitude’s value and were either positive or negative. To increase the amplitude of the EMF, positive offset values were set at +0.5 V, +1.5 V, +2.5 V, +4.0 V, and +10.0 V, respectively, for voltage stimulation at 1.0 V, 3.0 V, 5.0 V, 8.0 V, and 10.0 V. To decrease the amplitude of the EMF, negative offset values were set at -0.5 V, -1.5 V, -2.5 V, -4.0 V, and -10.0 V, respectively, for voltage stimulation at 1.0 V, 3.0 V, 5.0 V, 8.0 V, and 10.0 V.

During and after each EMF stimulation, the post-stimulation EMF data were analyzed in real time. If the sensor of interest (SOI) EMF pattern did not transform from a valley to a peak or show similar amplitude as that of the opposing sensor (OS), then the voltage was increased to the next level. For example, if post-stimulation EMF recording at 1.0 V showed the SOI remained at a valley, then the next EMF stimulation at 3.0 V was applied. The escalating dosage of voltage was applied until SOI’s EMF pattern showed a peak at the targeted frequency of interest. Simultaneously and after each EMF stimulation, clinical assessment was performed to assess whether each patient’s clinical symptoms such as headache or deficits such as right arm weakness improved.

## Results

The mean age of the 10 patients in this study was 47.1 years. Mechanisms of injury included spontaneous hypertensive intracranial hemorrhage (one patient) and head trauma after a motor vehicle collision (auto versus auto, auto versus motorcycle, and auto versus pedestrian), dirt bike accident, and ground-level fall (nine patients). Radiographic findings included spontaneous basal ganglia hemorrhage (one patient), isolated traumatic subdural hematoma (one patient), traumatic subarachnoid hemorrhage (one patient), and no intracranial abnormalities (seven patients). The targeted frequency of interest, voltage application, offset voltage value, and post-EMF stimulation interpretations are shown in Table 1. Improvement in EMF data was found in two patients after stimulation at 1.0 V, three patients after stimulation at 3.0 V, one patient after stimulation at 8.0 V, and two patients after stimulation at 10.0 V. Here, the authors use patient 5 as an illustrated example that showed improvement in EMF after the escalating dosage of stimulation.

**Table 1:** Electromagnetic field voltage application Hz, Hertz; SOI, sensor of interest; OS, opposing sensor

Patient	Targeted frequency of interest	Voltage (V) applied	Offset voltage value	Post-electromagnetic field stimulation interpretations
1	8.3 Hz	1.0 V	+0.5 V	After 1.0 V, SOI showed a peak and was at a higher amplitude than OS.
2	8.6 Hz	1.0 V	+0.5 V	After 1.0 V, SOI showed a similar amplitude as OS (positive slope).
3	7.7 Hz	1.0 V	+0.5 V	After 1.0 V, SOI remained at a valley.
		3.0 V	+1.5 V	After 3.0 V, SOI showed a peak and had a similar amplitude as OS.
4	7.3 Hz	1.0 V	+0.5 V	After 1.0 V, SOI remained at a valley.
		3.0 V	+1.5 V	After 3.0 V, SOI showed a peak, just like its OS.
5	7.6 Hz	1.0 V	+0.5 V	After 1.0 V, SOI was at a negative slope and had a lower amplitude than OS.
		3.0 V	+1.5 V	After 3.0 V, SOI showed a plateau and was at a lower amplitude than OS.
		5.0 V	+2.5 V	After 5.0 V, SOI showed a valley but was at a slightly higher amplitude than OS.
		8.0 V	+4.0 V	After 8.0 V, SOI showed a peak and was at a slightly lower amplitude than OS.
6	7.9 Hz	1.0 V	+0.5 V	After 1.0 V, SOI showed a negative slope and was at a similar amplitude as OS.
		3.0 V	+1.5 V	After 3.0 V, SOI showed a negative slope and was at a lower amplitude than OS.
		5.0 V	+2.5 V	After 5.0 V, SOI showed a negative slope and was at a slightly lower amplitude than OS.
		8.0 V	+4.0 V	After 8.0 V, SOI showed a negative slope and was at a slightly lower amplitude than OS.
		10.0 V	+5.0 V	After 10.0 V, SOI had a similar amplitude as OS.
7	8.7 Hz	1.0 V	+0.5 V	After 1.0 V, SOI showed a plateau and was at a lower amplitude than OS.
		3.0 V	+1.5 V	After 3.0 V, SOI showed a plateau and was at a lower amplitude than OS.
		5.0 V	+2.5 V	After 5.0 V, SOI showed a plateau and was at a lower amplitude than OS.
		8.0 V	+4.0 V	After 8.0 V, SOI showed a similar valley as OS and was at a lower amplitude than OS.
		10.0 V	+5.0 V	After 10.0 V, SOI showed a positive slope and was at a lower amplitude than OS. (A higher voltage needed?)
8	9.5 Hz	1.0 V	+0.5 V	After 1.0 V, SOI showed a negative slope and was at a lower amplitude than OS.
		3.0 V	+1.5 V	After 3.0 V, SOI showed a valley and was at a lower amplitude than OS.
		5.0 V	+2.5 V	After 5.0 V, SOI showed a negative slope and was at a lower amplitude than OS.
		8.0 V	+4.0 V	After 8.0 V, SOI showed a negative slope and was at a lower amplitude than OS.
		10.0 V	+5.0 V	After 10.0 V, SOI remained at a valley and was at a lower amplitude than OS. (A higher voltage needed?)
9	5.2 Hz	1.0 V	+0.5 V	After 1.0 V, SOI remained at a valley and at a lower amplitude than OS.
		3.0 V	+1.5 V	After 3.0 V, SOI showed a peak and was at a lower amplitude than OS.
10	10.4 Hz	1.0 V	+0.5 V	After 1.0 V, SOI showed a negative slope and was at a slightly higher amplitude than OS.
		3.0 V	+1.5 V	After 3.0 V, SOI showed a negative slope and was at a lower amplitude than OS.
		5.0 V	+2.5 V	After 5.0 V, SOI showed a negative slope and was at a lower amplitude than OS.
		8.0 V	+4.0 V	After 8.0 V, SOI showed a valley and was at a lower amplitude than OS.
		10.0 V	+5.0 V	After 10.0 V, SOI showed a negative slope and was at a similar amplitude as OS.

Illustrated case presentation: Patient 5

**Figure 1:**
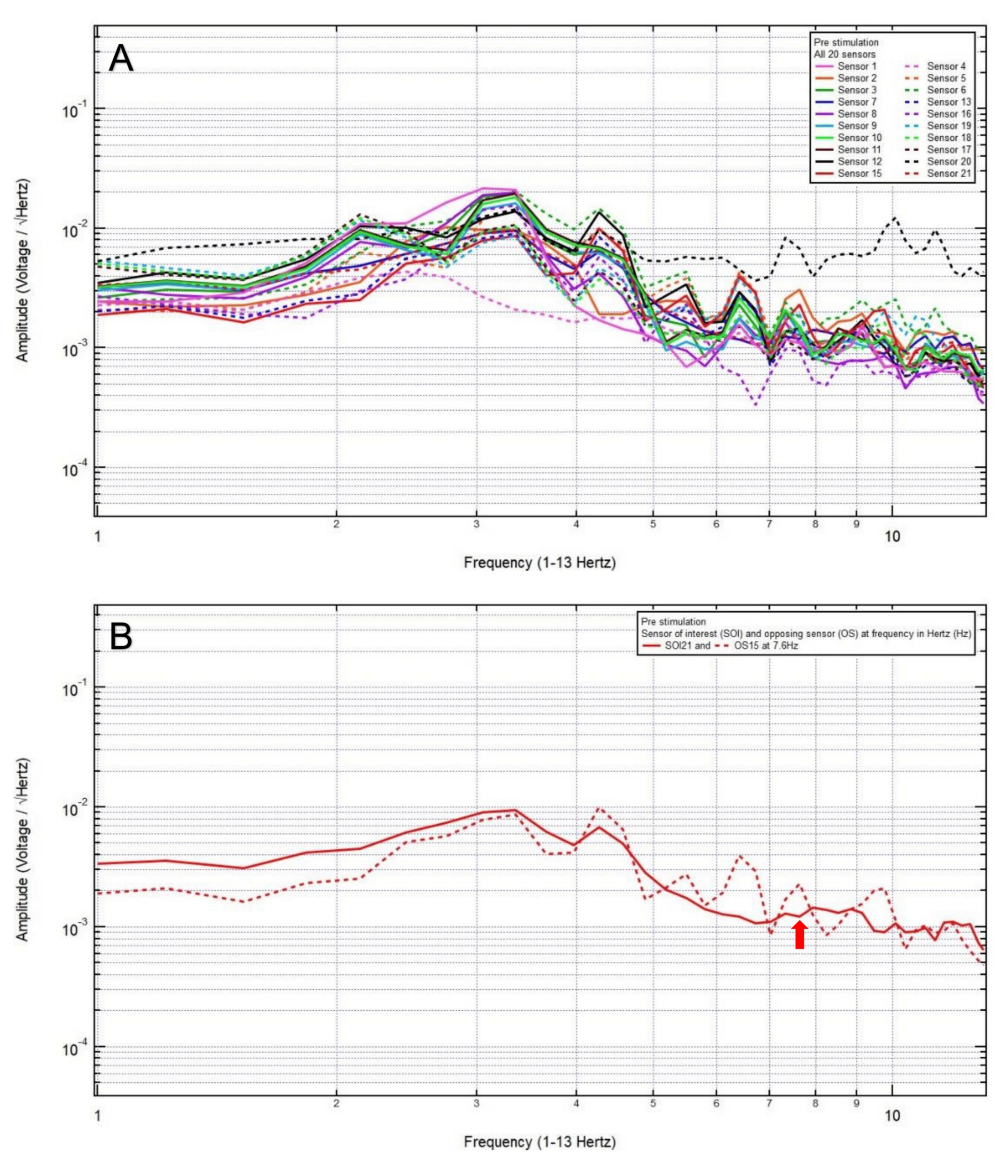
(A) Pre-stimulation electromagnetic field data of patient 5 showed the localization of the site of brain injury to sensor of interest 21 (left frontal lobe) with its opposing sensor 15 (left occipital lobe) and (B) revealed a pair of peak and valley at 7.6 Hertz (red arrow), which was selected as the frequency of interest

**Figure 2:**
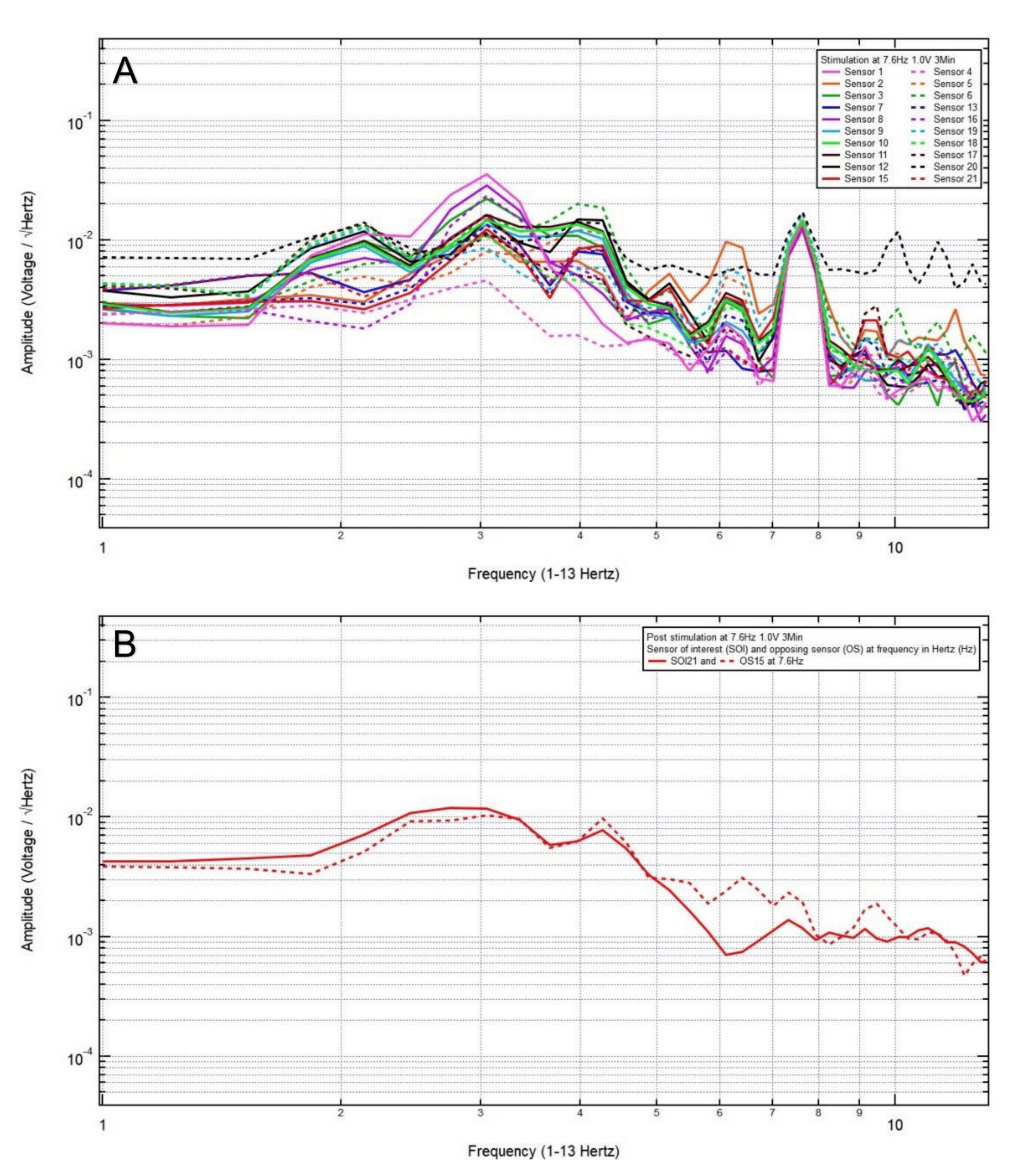
(A) Electromagnetic field stimulation at 7.6 Hertz and 1.0 voltage over three minutes was applied. (B) Post-stimulation electromagnetic field recording showed that at 7.6 Hertz, the sensor of interest was at a negative slope and at a lower amplitude than that of the opposing sensor (red arrow), suggesting that a higher voltage was needed

**Figure 3:**
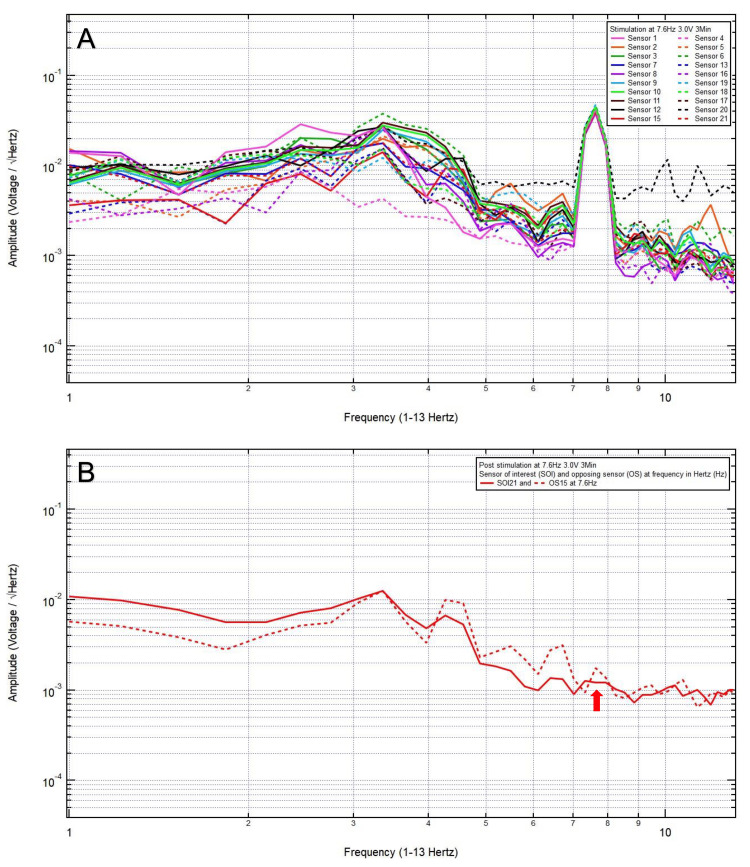
(A) Electromagnetic field stimulation at 7.6 Hertz and 3.0 voltage over three minutes was applied. (B) Post-stimulation electromagnetic field recording showed that at 7.6 Hertz, the sensor of interest was at a plateau and was at a lower amplitude than that of the opposing sensor (red arrow)

**Figure 4:**
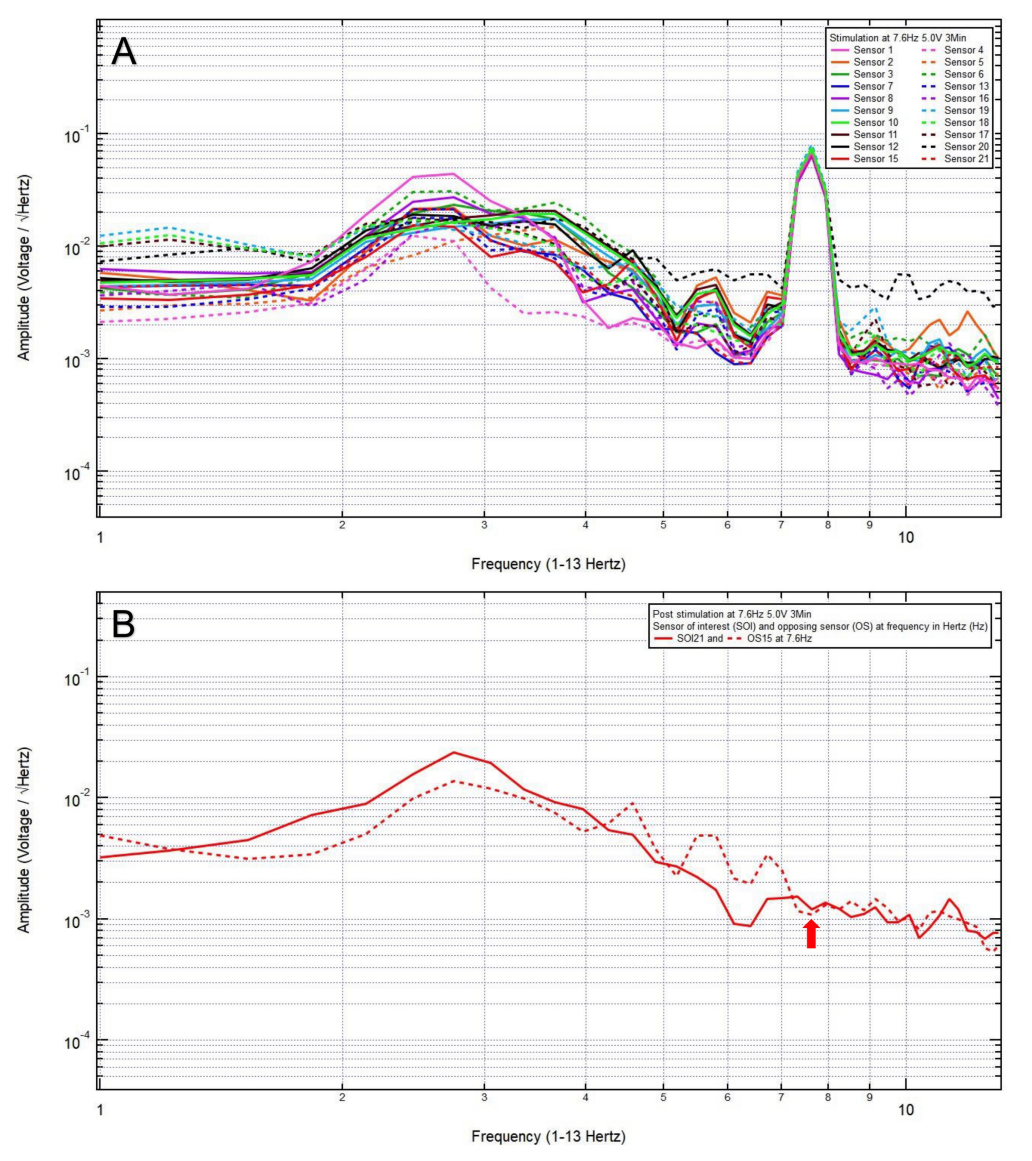
(A) Electromagnetic field stimulation at 7.6 Hertz and 5.0 voltage over three minutes was performed. (B) Post-stimulation electromagnetic field recording showed that at 7.6 Hertz, the sensor of interest was at a valley and at a slightly higher amplitude than that of the opposing sensor (red arrow)

**Figure 5:**
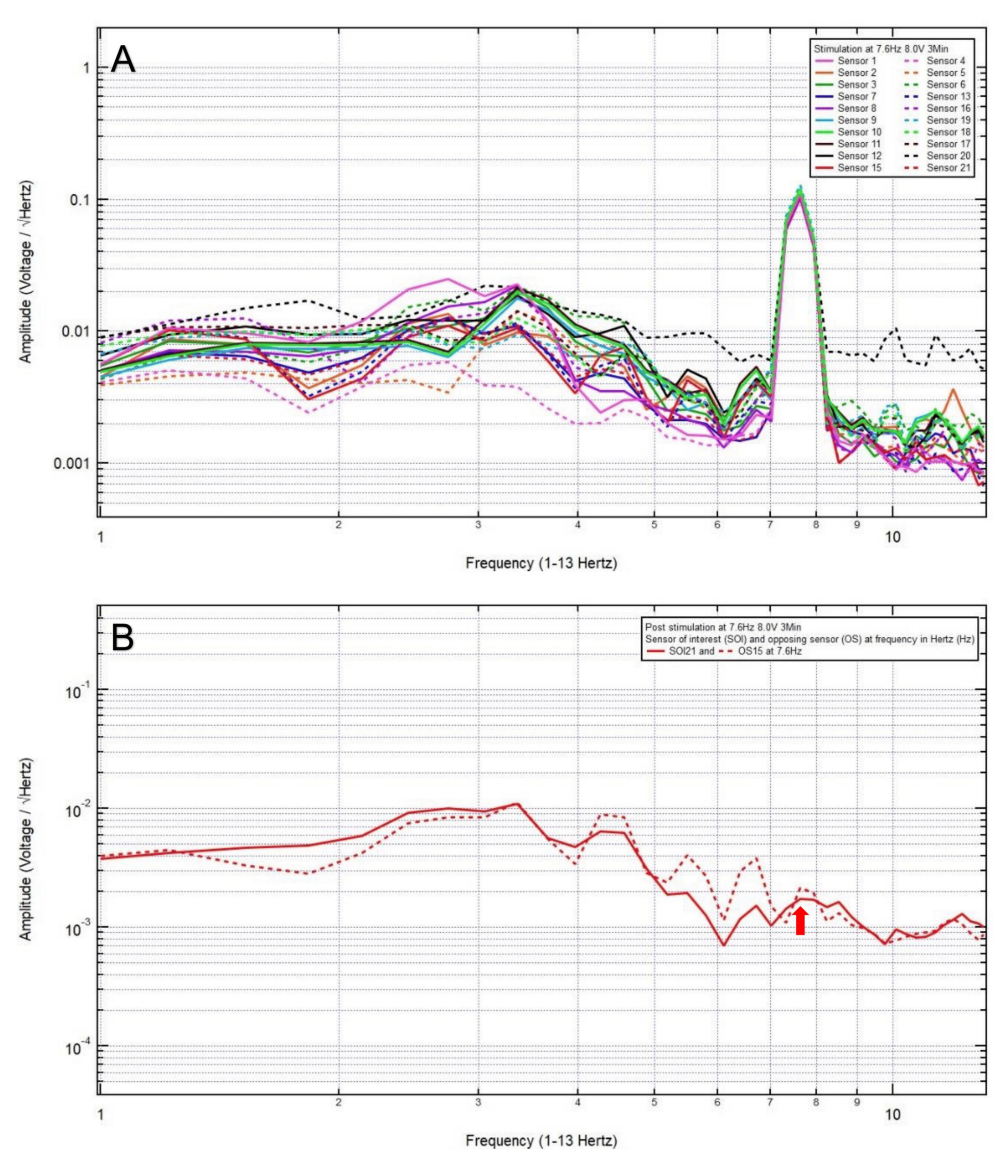
(A) Electromagnetic field stimulation at 7.6 Hertz and 8.0 voltage over three minutes was performed. (B) Post-stimulation electromagnetic field recording showed that at 7.6 Hertz, the sensor of interest was at a peak and at a slightly lower amplitude than that of the opposing sensor (red arrow)

An 81-year-old woman presented with a chief complaint of bilateral frontal headache, 1/10 in pain, after a ground-level fall. The patient was GCS15 with bilateral forehead ecchymosis. The patient’s pre-stimulation EMF data showed localization of the site of brain injury to SOI21 (left frontal lobe) with its OS15 (left occipital lobe) (Figure 1A) and revealed a pair of peak and valley at 7.6 Hz (red arrow), which was selected as the FOI (Figure 1B). EMF stimulation at 7.6 Hz and 1.0 V over three minutes was applied (Figure 2A), and post-stimulation EMF recording showed that the SOI was at a negative slope and at a lower amplitude than that of OS (red arrow), suggesting that a higher voltage was needed (Figure 2B). Therefore, EMF stimulation at 7.6 Hz and 3.0 V over three minutes was applied (Figure 3A), and post-stimulation EMF recording showed that the SOI was at a plateau and was at a lower amplitude than that of OS (Figure 3B). Next, EMF stimulation at 7.6 Hz and 5.0 V over three minutes was performed (Figure 4A), and post-stimulation EMF recording showed that the SOI was at a valley and at a slightly higher amplitude than that of OS (Figure 4B). Finally, EMF stimulation at 7.6 Hz and 8.0 V over three minutes was performed (Figure 5A), and post-stimulation EMF recording showed that the SOI was at a peak and at a slightly lower amplitude than that of OS (Figure 5B). Given that the last EMF recording showed a peak at the FOI, there was no indication to apply a higher voltage.

**Figure 6:**
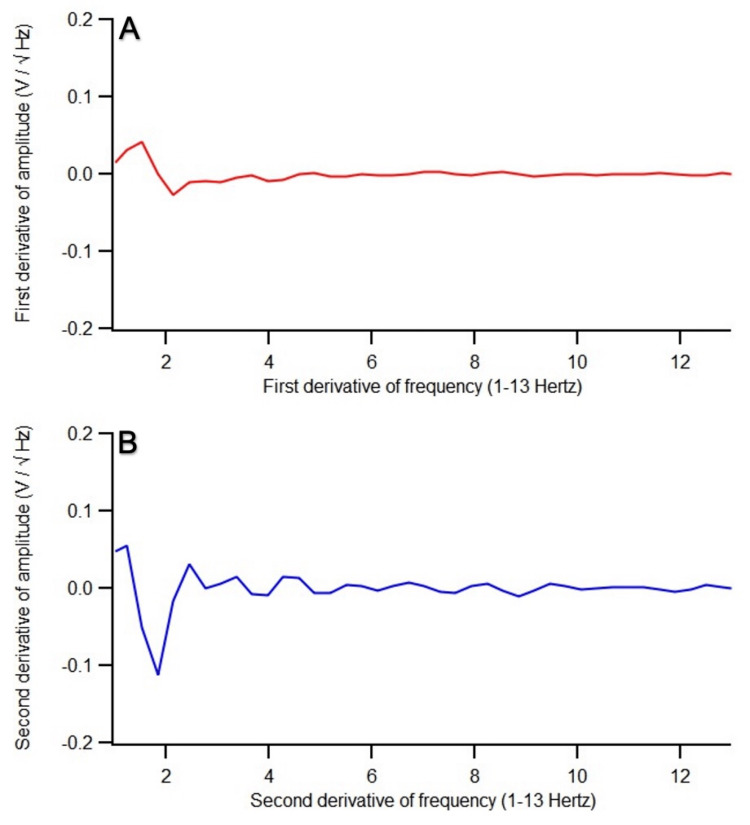
The (A) first derivative and (B) second derivative of the pre-stimulation EMF recording of patient 8 whose post-stimulation EMF recording at 10.0 voltage showed a valley There were much less defined waveforms and less fluctuations with overall fewer changes from peaks to valleys throughout 1.0-13.0 Hertz, especially after 5.0 Hertz V, voltage; Hz, Hertz; EMF, electromagnetic field

**Figure 7:**
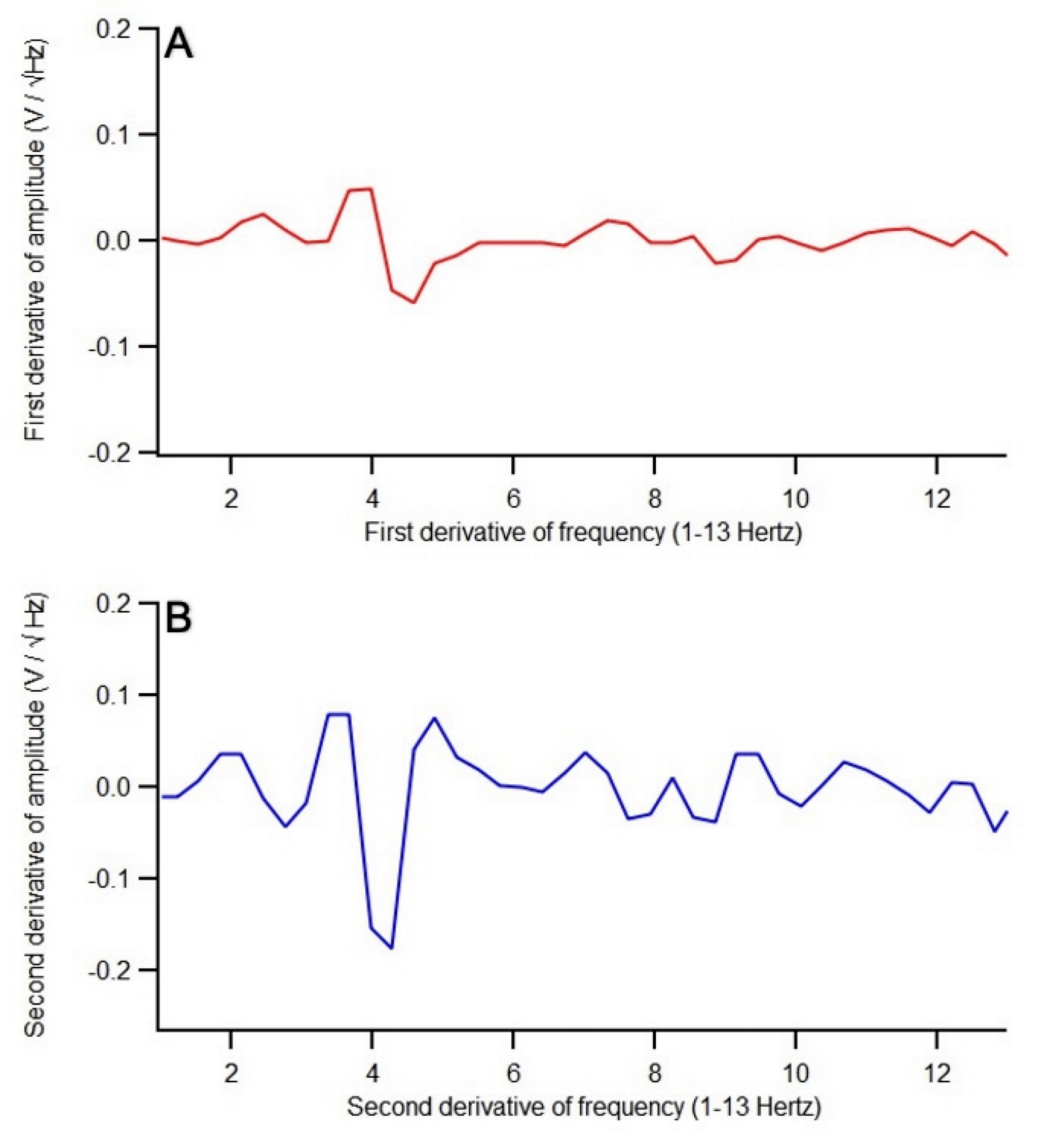
The (A) first derivative and (B) second derivative of the pre-stimulation EMF recording of patient 1 whose post-stimulation EMF recording at 1.0 voltage showed a peak There were much more defined waveforms and more fluctuations with overall more changes from peaks to valleys throughout 1.0-13.0 Hertz, especially after 5.0 Hertz V, voltage; Hz, Hertz; EMF, electromagnetic field

Two patients’ SOI post-stimulation EMF data did not show a peak or similar amplitude as that of the OS. Patient 7’s post-stimulation EMF recording at 10.0 V was beginning to show EMF changes; it was at a positive slope in the SOI, suggesting that if a higher voltage was given, for example, at 13.0 V, then the positive slope would likely have transformed into either a peak or similar amplitude as that of the OS. In this study’s protocol, the maximum voltage delivery was 10.0 V. On the other hand, patient 8’s post-stimulation EMF recording at 10.0 V showed that the SOI remained at a valley, also suggesting a higher voltage was needed. The first and second derivatives of the pre-stimulation EMF recording of this patient were analyzed (Figure 6A, 6B) and compared to those of patient 1 (Figure 7A, 7B). There were much less defined waveforms and less fluctuations with overall fewer changes from peaks to valleys throughout 1.0-13.0 Hertz in patient 8. Clinically, both patients reported improvement in headache severity. Headache is self-reported and is multifactorial. Clinical improvement in headache despite the SOI’s EMF data not showing as a peak or at a similar amplitude to that of the OS supports this.

## Discussion

This prospective study involved 10 patients with atraumatic and traumatic brain injury whose brain EMF was recorded, and EMF stimulation was applied within 24 hours of clinical presentation. Precise EMF stimulation was tailored to the abnormal brain frequencies from abnormalities of brain areas. The precise stimulation led to the modulation of abnormal EMF patterns, which became similar to the EMF patterns of normal brain activities. The escalating dosage of voltage stimulation was applied after identifying the abnormal brain frequencies until the amplitude of the SOI became either a peak or at a similar amplitude compared to that of the OS. This was achieved in eight out of 10 patients. In the other two patients, the neurons in the areas of brain injury may be sufficiently presently impaired such that 10.0 V may be ineffectual and may require a higher voltage. Patient 7’s EMF was beginning to show changes (valley into a positive slope), and improvement in EMF patterns likely can be achieved with the next level of voltage stimulation at 13.0 Hz. This was not performed because the study’s protocol capped the maximum dosage at 10.0 V. On the other hand, for patient 8, his post-stimulation EMF data at 10.0 V showed a valley. Perhaps this patient at baseline had a less “healthy” brain as defined by fewer fluctuations in waveforms as previously described by Brazdzionis et al. and therefore would require higher voltage stimulation to induce EMF changes [7]. Indeed, in comparison to another patient (patient 1) whose post-stimulation EMF at 1.0 V showed a peak, there were much less defined waveforms and less fluctuations with overall fewer changes from peaks to valleys throughout 1.0-13.0 Hertz in patient 8’s first and second derivatives from SOI11 (Figure 6A, 6B), whereas there were much more defined waveforms and more fluctuations with overall more changes from peaks to valleys throughout 1.0-13.0 Hertz in patient 1’s first and second derivatives from SOI20 (Figure 7A, 7B). This finding is consistent with previous findings where there was less variability in EMF measurements in patients with neural pathology, which may be correlated with disrupted neural signaling [7]. Less variability suggests more disruptions in the neural circuitry that would likely require a higher voltage to achieve normal realignment of the electrochemical signaling among the neurons. The derivatives of pre-stimulation EMF provide insight into which patients would likely need an escalating dosage of voltage stimulation.

The way this study used EMF recordings is different from how other technologies such as PEMF, ELF-MF, and magnetoencephalography (MEG) are used. In this study, the EMF recordings were collected and analyzed in real time. This allowed the immediate assessment of brain activity and the determination of the next step in treatment. For example, if the post-EMF stimulation did not show improvement, then a higher voltage was delivered. The real-time feedback of the EMF recordings determined whether to escalate the dosage of voltage stimulation. The goal of EMF stimulation is to transform abnormal EMF patterns back to those of the EMF patterns seen over brain regions without injury because this transformation reflects the modulation of the neuronal circuitry at the molecular and cellular levels. In a pilot study using a Yucatan miniswine model of TBI with and without EMF stimulation, several genes were found to be differentially expressed: *INSC*, *TTR*, *CFAP126*, *SEMA3F*, *CALB1*, *CDH19*, and *SERPINE1*, and they were associated with immune cell infiltration, myelination, reactive oxygen species regulation, thyroid hormone transportation, cell proliferation, and cell migration [1]. The neuroprotective and regenerative effects of EMF stimulation included the decreased occurrence of neural apoptosis and increased levels of neuron-specific enolase, which is involved in neurite regeneration [3].

The modulation of brain activity using EMF has been previously studied. In animal studies, PEMFs have been shown to reduce the size of the infarcted area of stroke and decrease pro-inflammatory mediators [10]. PEMF upregulates A2A and A3 adenosine receptors, counteracts hypoxia-induced apoptosis and reactive oxygen species production, and has an anti-inflammatory effect on microglial cells [11-14]. In human studies, PEMFs have been shown to have a dose-dependent reduction in the lesion size of acute ischemic stroke [15]. PEMFs appear to provide neuroprotection and reduce functional deficits following acute ischemic stroke [16]. A detailed review of the effects of PEMF on the brain can be found in Flatscher et al.’s study [17]. Unlike PEMF, which delivers EMF stimulation in pulses as the name suggests, the EMF stimulation in this study was delivered continuously. The EMF applied should be able to reproduce the PEMF effects when a similar frequency, waveform, and voltage are used. However, EMF can affect many more functions when specific to the patient or cellular signals are known. This technology is available to identify specific brain abnormal EMF signals at various frequencies and attempt to change those frequencies in real time through monitoring the EMF pattern and the patient’s neurological signs and symptoms.

Unlike MEG, which is a diagnostic tool, the helmet and sensor setup in this study makes it both a diagnostic and therapeutic tool. The MEG is a non-invasive test that measures brain function and maps the findings onto magnetic resonance imaging to produce magnetic source imaging. MEG is used in preoperative brain mapping and epilepsy surgery [21]. MEG only records the magnetic activity of the brain and does not record the electrical activity. On the other hand, the helmet and sensor setup in this study measures both the electrical field and the magnetic field, aka the electromagnetic field. The EMF recording is accomplished using a continuous, non-invasive, noncontact helmet and sensors through the entire brain as the brain generates changes in its intrinsic EMF [7,18-20]. The helmet and sensor setup are brought to the patient in their room, the clinic office, and even the ambulance, which differs from a patient going to a room where the MEG setup is. The advantage of being portable is that it allows for the increased utility of this technology, as some patients may be hemodynamically unstable to be brought to the MEG room. Moreover, the sensors are connected to a stimulator box that allows for the delivery of magnetic fields to modulate brain activities, making it a therapeutic tool. The ability to diagnose and treat abnormal EMF patterns precisely and in real time via a non-invasive portable helmet demonstrates the potential to widely perform EMF diagnosis and treatment on diverse neurosurgical diseases and facilitate the restoration of normal brain communication.

Limitations

First, the small sample size limits generalizability. Second, there is no follow-up on these patients to allow for the evaluation of the long-term effects of EMF modulation. Third, any clinical changes may be induced by a placebo effect. Further studies with larger cohorts and randomized controlled trials may aid in validating this study’s findings, investigating risk factors for higher-voltage stimulation, and studying the long-term effects of EMF modulation via stimulation.

## Conclusions

The modulation of the brain at the optimal frequency and voltage stimulation was correlated with improvements in clinical symptoms and signs. The applied EMF frequency was precisely tailored to the identified EMF brain abnormalities of each patient. The escalating dosage of voltage stimulation was followed in real time until the optimal voltage stimulation, one that transformed abnormal EMF patterns into normal EMF patterns, was found. Modulating the brain this way likely reversed the malfunction of molecular and cellular changes and facilitated the recovery of brain functions.
